# Impact of Social Identity Complexity in Unfair Events on Intergroup Bias in Third-Party Fairness Maintenance

**DOI:** 10.3390/bs13060456

**Published:** 2023-06-01

**Authors:** Zhuang Li, Gengdan Hu, Qiangqiang Li

**Affiliations:** 1 Department of Psychology, School of Humanities, Tongji University, Shanghai 200092, China; 2 Clinical Research Center for Mental Disorders, Shanghai Pudong New Area Mental Health Center, School of Medicine, Tongji University, Shanghai 200124, China; 3 School of Educational Science, Jiangsu Second Normal University, Nanjing 210013, China

**Keywords:** third-party fairness maintenance, intergroup bias, ingroup love, outgroup hate, social identity complexity

## Abstract

The intergroup bias in third-party fairness maintenance includes two components: ingroup love and outgroup hate. Previous studies revealed that intergroup bias could be alleviated by high social identity complexity. This study explored the influence of the social identity complexity of parties in unfair events on intergroup bias in third-party fairness maintenance. Participants were divided into two groups and asked to choose from retention and punishment (Experiment One)/compensation (Experiment Two) to respond to unfair events presented by dictator games. To separate the components, we introduced additional unaffiliated members. Social identity complexity included single identity, presented as ingroup vs. outgroup sides of unfair events, and multiple identities, which included group identity and five additional identities. The results demonstrated that third parties tended to impose less punishment and more compensation on outgroup members under multiple than single identity conditions; however, the punishment and compensation to ingroup members exhibited no significant difference between the identity conditions. These results indicated that the multiple identities of the two sides in unfair events can reduce intergroup bias in third-party fairness maintenance, which can be achieved by reducing the outgroup hate rather than ingroup love.

## 1. Introduction

### 1.1. Ingroup Love and Outgroup Hate in Intergroup Bias

**Figure 1:**
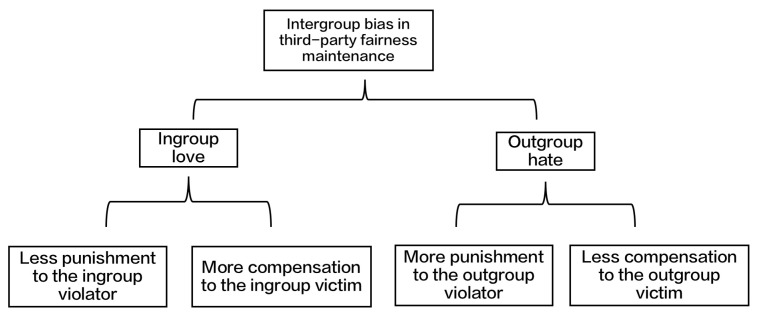
Ingroup love and outgroup hate in third-party fairness maintenance.

Fairness is an important social norm. To maintain fairness, people intervene in unfair events unrelated to themselves, which is referred to as third-party fairness maintenance [1]. Third-party fairness maintenance includes two methods: punishment, which targets the violators in unfair events, and compensation, which targets the victims [2]. Numerous studies have demonstrated that third-party fairness maintenance contains intergroup bias, which is composed of ingroup love and outgroup hate [3,4,5,6,7]. When the violator is an ingroup member, the third party tends to be more tolerant, and the punishment is less severe; however, when the victims are ingroup members, greater compensation than punishment is provided, which could be considered ingroup love. Conversely, when the violator is an outgroup member, the third party tends to be harsher, and the punishment severer; however, when the victim is an outgroup member, the compensation is lower, which is considered outgroup hate (see Figure 1).

Intergroup bias exists in social interactions and has been extensively studied by scholars; however, whether ingroup love and outgroup hate coexist has been disputed. Social identity theory argues that individuals perceive internal and external groups as competing [8]. When intergroup conflict occurs, individuals act to damage the external group interests and expand the internal group interests to keep their comparative advantages [9,10]. However, the realistic conflict theory holds that intergroup conflict only promotes individuals to pursue the maximization of absolute intergroup interests rather than comparative advantages [11,12]. Therefore, individuals expand internal group interests but do not deliberately damage external group interests [13]. As such, social identity theory suggests that the two components coexist, whereas the realistic conflict theory states that they do not.

Studies have used the intergroup prisoner’s dilemma game to observe whether individuals would participate in intergroup competition at the expense of personal interests. Participants were offered to retain personal interests but not participate in intergroup competition or to sacrifice personal interests to promote ingroup interests while damaging outgroup interests; the participants tended to choose the latter [14,15]. This response indicated that the two components coexisted and supported the opinion of the social identity theory [8]. Studies that added a third option to sacrifice personal interests to promote ingroup interests without damaging outgroup interests found that individuals tended to choose the new option [16,17,18,19]. This finding indicated that the two components were independent and supported the opinion of realistic conflict theory. Allport, the representative of experimental social psychology, also held the same viewpoint [20]. Brewer and Campbell investigated the prosocial mentality of more than 30 tribes in East Africa and found that almost all tribes gave a positive evaluation of the internal group in terms of trust, friendship, and honesty; however, this positive evaluation was not associated with the social distance to the outgroup [21]

From the research on the intergroup prisoner’s dilemma, we can see that whether the two components can be separated in the research method will affect our observation of the relationship between the two. However, few existing studies rarely separate the two components when discussing intergroup bias in third-party fairness maintenance. Only Schiller combined the prisoner’s dilemma game with third-party punishment and introduced unaffiliated members who were neither from an internal nor external group [22]. In the game, the betrayers (violators) were ingroup members, unaffiliated members, and outgroup members; the collaborators (victims) were ingroup members. The results demonstrated that third-party punishment was severer for outgroup violators than unaffiliated members, indicating outgroup hate, whereas punishment for ingroup violators was milder, indicating ingroup love. Thus, the introduction of unaffiliated members could effectively separate the two components. However, an important deficiency of Schiller’s research is that it focused on punishment but not compensation.

### 1.2. The Impact of Social Identity Complexity on Intergroup Bias

Intergroup bias creates intergroup conflict, which damages social stability. Therefore, it is important to consider controlling intergroup bias. Social complexity may provide a reference. In social life, individuals usually have multiple social identities, and social identity complexity refers to the mental representation of the degree of overlap of their multiple identities [23]. A higher degree of overlap indicates lower social identity complexity. Conversely, a lower degree of overlap indicates higher social identity complexity [24].

Roccas and Brewer measured the degree of overlap of four social identities (nation, race, religion, and university) and closeness to outgroup members among American college students and found that higher social identity complexity was associated with higher closeness [23]. Roccas and Brewer analyzed the relationship between social identity complexity and acceptance of Soviet immigrants among Israeli college students and revealed that higher social identity complexity was associated with higher acceptance [23]. Brewer and Pierce found that individuals with higher social complexity residing in Ohio, US, had more positive attitudes toward multiculturalism and anti-discrimination movements and a higher tolerance for minorities [25]. Schmid investigated the relationship between intergroup contact, social identity complexity, and intergroup attitude based on religious beliefs in Northern Ireland and found that more intergroup contact increased social identity complexity, reduced intergroup bias, and improved tolerance of external groups, with social identity complexity playing a mediating role [26]. Therefore, social identity complexity could modify individuals’ intergroup attitudes and alleviate intergroup bias.

Social identity complexity includes not only an individual’s subjective mental representation of their multiple social identities but also the objective social identity complexity of others [27]. Unlike subjective social identity complexity, objective social identity complexity is reflected by identity quantity. A greater number of identities is associated with more objective social identity complexity, which has a positive impact on intergroup relationships. Xin and Xin found that people with greater social identity are more easily trusted by others. Social distance plays a mediating role between trust and social identity complexity, which is moderated by intergroup relationships. Specifically, multiple identities can only enhance an individual’s trust in outgroup members but do not affect the trust in ingroup members [27]. This tendency indicates that other people’s objective social identity complexity urges individuals to modify their attitude toward outgroup members but does not affect their attitude toward ingroup members. Similar to the study by Xin and Xin, the present study also focused on the objective social identity complexity of the two parties in unfair events rather than subjective mental social identity complexity.

### 1.3. The Present Study

The third party is not a party to an unfair event and is less affected by emotion and self-interest when dealing with the unfair event. Therefore, it is more objective and impartial in maintaining fairness [28]. However, intergroup bias greatly reduces the impartiality of third-party fairness maintenance. Unfair events damage fair norms. Third-party fairness maintenance that is subject to intergroup bias further damages fair norms. Therefore, exploring ways to eliminate intergroup bias in third-party fairness maintenance is of great significance in promoting social equity.

Social identity complexity modifies intergroup attitudes and alleviates intergroup conflicts. This study investigated whether this effect exists in third-party fairness maintenance and, if so, how it affects ingroup love and outgroup hate. This study posed the following four questions:

(1) Can multiple identities of the two sides of unfair events reduce intergroup bias in third-party fairness maintenance?
(2) If so, is this result achieved by reducing outgroup hate, ingroup love, or both?
(3) If it is achieved by reducing ingroup love, does this enhance the punishment for ingroup violators, reduce compensation for ingroup victims, or both?
(4) If it is achieved by reducing outgroup hate, does this reduce punishment for outgroup violators, enhance compensation for outgroup victims, or both?

## 2. Experiment One: Impact of Social Identity Complexity on Intergroup Bias in Third-Party Punishment

In Experiment One, we adopted the method of Schiller to introduce unaffiliated members to ingroup and outgroup members to separate ingroup love and outgroup hate. We aimed to explore the impact mechanism of multiple identities on intergroup bias in third-party fairness maintenance through punishment tasks. In Experiment Two, we presented unfair events through the dictator game. Dictators (equivalent to violators) were ingroup, outgroup, and unaffiliated members, and recipients (equivalent to victims) were unaffiliated. In the face of unfair events, the third-party chose between retention and punishment. If the punishment to the ingroup dictator was less than that accorded to the unaffiliated dictator, it was an indication of ingroup love. If the punishment to the outgroup dictator was more than that accorded to the unaffiliated dictator, it was an indication of outgroup hate. If the punishment to the outgroup dictator was no longer more than that accorded to the unaffiliated dictator, it meant that the outgroup hate decreased or disappeared. If the punishment to the ingroup dictator was no longer less than that accorded to the unaffiliated dictator, it meant that the ingroup love had decreased or disappeared. It should be noted that in Schiller’s study, the victims were ingroup members, resulting in more severe punishment. In this study, however, we wanted to discuss a pure punishment, so the victims were unaffiliated.

### 2.1. Participants

We recruited 87 college students from Tongji University, including 46 men aged 17–24 years, with an average age of 20.16 ± 1.86 years. When recruiting participants, we collected their identity information in five aspects (described below).

### 2.2. Procedure

#### 2.2.1. Collect and Determine Social Identities

Before the formal experiment, we collected and determined the multiple identities using questionnaires and measurements to determine specific identities to represent social identity complexity.

First, 230 questionnaires were distributed to college students at Tongji University to collect social identities. The questionnaire posed two questions: (1) When you introduce yourself to others, which identities will you demonstrate? (2) When meeting a stranger, what identities do you want to know? The questionnaire did not limit the number of responses. The aim was to collect as many identities as possible. In total, 201 valid questionnaires were collected, and 13 identities were preliminarily obtained: hometown, graduate college, major, hobbies, occupation role, constellation, family background, workplace, political party, gender, educational background, registered residence (rural or urban), and marriage and relationship status.

Next, we obtained ratings of the importance score of social identities. In total, 175 questionnaires were distributed to college students at Tongji University. We asked the participants to rate the importance of the above 13 identities using a forward direction score of 1–7 points. In total, 169 valid questionnaires were collected. The overall average score of the 13 identities was 4.40 ± 1.71. The following identities had higher average scores than the overall score: hometown (5.86 ± 1.74), college (5.82 ± 1.81), major (5.60 ± 1.32), hobbies (95.62 ± 1.16), occupation (4.66 ± 1.35), constellation (4.45 ± 1.27), and family background (4.41 ± 1.25). As the participants were from the same university, the identity of “college” was not distinguishable; therefore, we changed the category to “department”. In addition, as the participants were all college students, we discarded the identity of “occupation”. Finally, as “family background” was too general, we modified it to “parental occupation” in combination with the student status of the participants. Thus, six social identities were identified: hometown, college, major, hobbies, constellation, and parents’ occupation.

When we recruited participants in the formal experiment, we required them to provide these six identifiers. However, some participants did not know their constellations; therefore, we discarded this identity and used the remaining five in the final study.

#### 2.2.2. Grouping

The participants were grouped through the minimal group paradigm. They were given a pack of sticky notes in two colors (yellow and blue). The participants freely chose the color they liked and were divided into two groups: group A (yellow, 47 people) and group B (blue, 40 people). Every participant was assigned an ID number.

#### 2.2.3. Dictator Game

The participants took part in this phase as dictators. The dictator game was conducted for two reasons: as a group identity operation test (see below) and to familiarize participants with the procedure of the dictator game to prepare for the subsequent third-party fairness maintenance. The participant (dictator) was taken into a room with a computer and told that there was another participant (recipient) in another room. The dictator had to allocate 100 tokens to the recipient. Each token corresponded to 0.1 RMB (approximately USD 0.015). We presented the ID number and group identity of the recipient to the dictator through a computer. Participants were told that some other participants were late and did not participate in the grouping process; thus, they would participate in the dictator game as unaffiliated members. The participants were dictators, and the recipients were ingroup, outgroup, and unaffiliated members. Each recipient’s identity was presented four times. The dictator game had 12 trials, and they were presented randomly. There were nine allocation schemes for participants to choose from in each round: 90:10 (dictator gets 90 tokens and gives 10 tokens to the recipient), 80:20, 70:30, 60:40, 50:50, 40:60, 30:70, 20:80, and 10:90. In fact, there was no other participant playing as the recipient, and the twelve-trial game was the pre-set computer program. After the dictator game was over, we randomly selected one trial and calculated the test fee for the phase of the dictator game according to the choice of the participant.

#### 2.2.4. Third-Party Fairness Maintenance

The participants were third parties in this phase. The dictator game was followed by a break and then third-party fairness maintenance. The participant was told that two people were playing the dictator game in the other room, and the game rules were the same as the rules of the dictator game that just ended. We presented the dictator game results to the participants through a computer and showed the group identities of the two sides of the game.

The recipients were all unaffiliated members, whereas the dictators were ingroup, outgroup, and unaffiliated members. There were four allocation results: 90:10, 80:20, 70:30, and 60:40, and each scheme was presented three times. Thus, there were 36 trials (three dictators’ group identities × 4 allocation results × 3 present times), and they were presented randomly. In reality, the dictator game did not happen, and the computer presented a pre-designed program.

The participants were equally assigned to two types of social identity complexity treatment: single identity (44 persons) and multiple identities (43 persons). In the former, we only presented the group identity of dictators. However, in the latter, in addition to the group identity, we presented five other identities of the dictators. The participants were asked to rate the dictator’s social identity complexity in the range of 1–7. Higher scores indicated higher social identity complexity. In fact, the five-identity information was randomly selected and composed from identity information registered when we recruited the participants.

In this phase, the participant had an initial endowment of 50 tokens in each trial. In the face of unfair dictator game results, participants were asked to choose between retaining the situation and punishment. If retaining was selected, all 50 tokens would belong to participants. If punishment was selected, the number of tokens for punishment needed to be further filled in. The amount for punishment was taken from the 50 tokens, which meant that choosing punishment would result in losses to the third party. The punishment efficiency was 1:3, which meant that one token for punishment would cause a three-token loss to the dictator. To avoid the dictator’s token amount becoming negative, we stipulated that the number of tokens belonging to the dictator should at least be 0. After the experiment, we randomly selected one trial and calculated the test fee according to the participant’s choice. The final test fee was the sum of the test fees of the two phases of the dictator game and third-party fairness maintenance.

### 2.3. Results

#### 2.3.1. Group Identity Operation Test

Repeated measures ANOVA were used to compare the average number of tokens allocated to recipients in different groups. The independent variable was the recipients’ group identity comprising three levels: ingroup, outgroup, and unaffiliated. The participants assigned the highest average amount to ingroup recipients (51.81 ± 14.65), followed by unaffiliated recipients (41.78 ± 12.04) and outgroup recipients (33.07 ± 12.40). The difference among the three was significant: *F* (2,85) = 144.53, *p* < 0.001, η^2^p = 0.63. The post hoc test showed that the difference between the first two levels was significant (*p*_s_ < 0.001). Friedman’s rank test showed that the average ranges for ingroup, unaffiliated, and outgroup were 2.84, 1.96, and 1.20, χ^2^(2) = 132.96, *p* < 0.001. This result indicated that the participants had an intergroup bias, and the group identity operation was effective.

#### 2.3.2. Social Identity Complexity Operation Test

We used an independent *t*-test to compare the average score of identity complexity under the condition of two kinds of social identity complexity. The results showed that the multiple identity treatment (4.64 ± 0.85) was significantly higher than the single identity treatment: (2.44 ± 0.85), t (85) = 12.08, *p* < 0.001, Cohen’s d = 2.59. The Mann-Whitney U test showed that the average range for single-identity treatment was 24.40, and for multiple-identity treatment, it was 64.06, Z = −7.325, *p* < 0.001. Thus, the operation of social identity complexity was effective.

#### 2.3.3. Punishment Frequency and Amount

**Figure 2:**
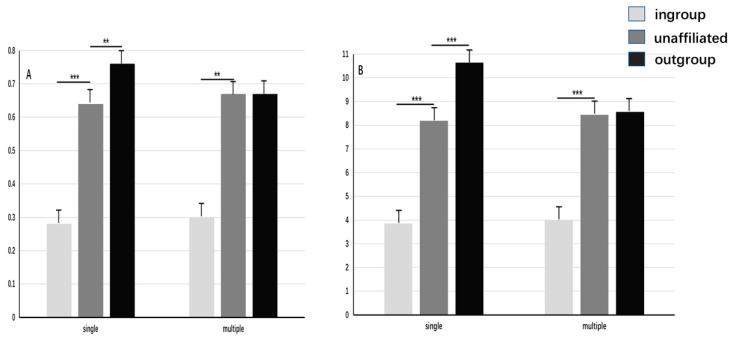
Impact of social identity complexity on intergroup bias in third-party fairness punishment. Note. (**A**) = punishment frequency. (**B**) = punishment amount. *** *p* < 0.001, ** *p* < 0.01. The error bars are standard errors.

We used a repeated measure ANOVA of 3 (dictator’s group identity: ingroup, outgroup, unaffiliated; within) × 2 (social identity complexity: single, multiple; between) to compare the punishment frequency. Punishment frequency was calculated by dividing punishment times by 12 [28,29] (see Table 1). The results showed that the main effect of the dictator’s group identity was significant: *F* (2,85) = 178.62, *p* < 0.001, η^2^p = 0.68. The punishment frequency for the outgroup dictator was higher than that for the unaffiliated dictator, and the latter was significantly higher than that for the ingroup dictator (*p*_s_ < 0.001). The main effect of social identity complexity was not significant: *F* (1,85) = 0.25, *p* = 0.62. The interaction effect was significant: *F* (2,85) = 3.82, *p* = 0.024, η^2^p = 0.043. The simple effect test showed that in the single treatment, the punishment frequency for the outgroup dictator was significantly higher than that for the unaffiliated dictator and ingroup dictator (*p*_s_ < 0.001), and the punishment frequency for the unaffiliated dictator was significantly higher than that for the ingroup dictator (*p* < 0.01). In the multiple identities’ treatment, the punishment frequency for the outgroup dictator remained significantly higher than that for the unaffiliated dictator and ingroup dictator (*p*_s_ < 0.01); however, the difference between the latter two was no longer significant (*p* = 0.97; see Figure 2).

We used the same method of repeated measures with ANOVA to compare the punishment amount, which was calculated by dividing the number of tokens used for punishment by 12 [28,29] (see Table 1). The results showed that the main effect of a dictator’s group identity was significant: *F* (2,85) = 147.36, *p* < 0.001, η^2^p = 0.63. The average amount of punishment for the outgroup dictator was higher than that for the unaffiliated dictator (*p* < 0.001), and the latter was significantly higher than that for the ingroup dictator (*p* < 0.001). The main effect of social identity complexity was not significant: *F* (1,85) = 2.79, *p* = 0.10. The interaction was significant: *F* (2,85) = 7.21, *p* < 0.001, η^2^p = 0.08. The simple effect test showed that in the single identity condition, the average amount of punishment for the outgroup dictator was significantly higher than that for the unaffiliated dictator and ingroup dictator (*p*_s_ < 0.001), and the average amount of punishment for the unaffiliated dictator was significantly higher than that for the ingroup dictator (*p* < 0.001). In the multiple identities’ treatment, the punishment amount for the outgroup dictator remained significantly higher than that for the unaffiliated dictator and ingroup dictator (*p*_s_ < 0.01); however, the difference between the latter two was no longer significant (*p* = 0.96, see Figure 2).

The results of Experiment One showed that the third party punished the outgroup dictator more severely than the unaffiliated dictator; it punished the unaffiliated dictator more severely than the ingroup dictator. The former was an indication of outgroup hate, and the latter was an indication of ingroup love. Moreover, the results demonstrated that multiple social identities reduced the punishment for the outgroup dictator but did not reduce the punishment for the ingroup violator. This result indicated that multiple identities could reduce outgroup hate and decrease intergroup bias, whereas ingroup love was not affected by multiple identities. However, with multiple identities, the punishment for outgroup members remained higher than for ingroup members. Thus, we infer that intergroup bias can be reduced by multiple identities by reducing outgroup hate but cannot be eliminated. We verified this conclusion using a third-party compensation task in Experiment Two.

## 3. Experiment Two: Impact of Social Identity Complexity on Intergroup Bias in Third-Party Compensation

In Experiment Two, the dictators were unaffiliated members, and the recipients were ingroup, outgroup, and unaffiliated members. The third-party made a choice between retention and compensation. Experiment Two aimed to explore the impact mechanism of social identity complexity on intergroup bias in third-party fairness maintenance through the compensation task.

### 3.1. Participants

We recruited 85 college students from Tongji University, including 39 men aged 17–23 years, with an average age of 20.34 ± 1.70 years.

### 3.2. Procedure

The participants were divided into two groups, A (46 persons) and B (39 persons), by using the simple group paradigm, which was the same as in Experiment One. Then, the dictator game was played, which was the same as in Experiment One.

Next, we conducted third-party fairness maintenance. We presented unfair events through unfair results of the dictator game, which was the same as in Experiment One and was a preset program by computer. Dictators were unaffiliated members, and recipients were ingroup, outgroup, and unaffiliated members. As in Experiment One, the results of the dictator game were four unfair allocation schemes, each of which was presented thrice, with 36 trials in total. Faced with the unfair result, the third party chose between retaining and compensation (3 recipient group identities × 4 allocation results × 3 present times). The initial endowment, compensation efficiency, and calculation method of the test fee were the same as those in Experiment One. The participants were equally assigned to two social identity complexity treatments: single identity (42) and multiple identities (43). In the former, only the group identity of recipients was presented. In the latter, five other social identities, which were the same as in Experiment One, were presented. The participants were asked to rate the scores of social identity complexity of recipients, and the rating method was the same as in Experiment One.

### 3.3. Results

#### 3.3.1. Group Identity Operation Test

The test method was the same as in Experiment One. The number of tokens allocated to the recipients from high to low was ingroup, unaffiliated, and outgroup. The difference was significant: *F* (2,83) = 176.67, *p* < 0.001, η^2^p = 0.67. The post hoc test showed that the difference between each pair was significant (*p*_s_ < 0.001). Friedman’s rank test showed that the average ranges for ingroup, unaffiliated, and outgroup were 2.83, 1.98, and 1.19, χ^2^(2) = 122.43, *p* < 0.001. This result indicated that the participants had an intergroup bias, and the group identity operation was effective.

#### 3.3.2. Social Identity Complexity Operation Test

The test method was the same as in Experiment One. The results showed that the score of social complexity in multiple identities treatment (4.72 ± 0.58) was significantly higher than that in the single identity treatment: (2.70 ± 0.50), *t* (83) = 17.23, *p* < 0.001, Cohen’s *d* = 3.73. The Mann-Whitney U test showed that the average range for single-identity treatment was 21.69, and for multiple identity treatment, it was 63.81, Z = −7.87, *p* < 0.001. Thus, the operation of social identity complexity was effective.

#### 3.3.3. Compensation Frequency and Amount

**Figure 3:**
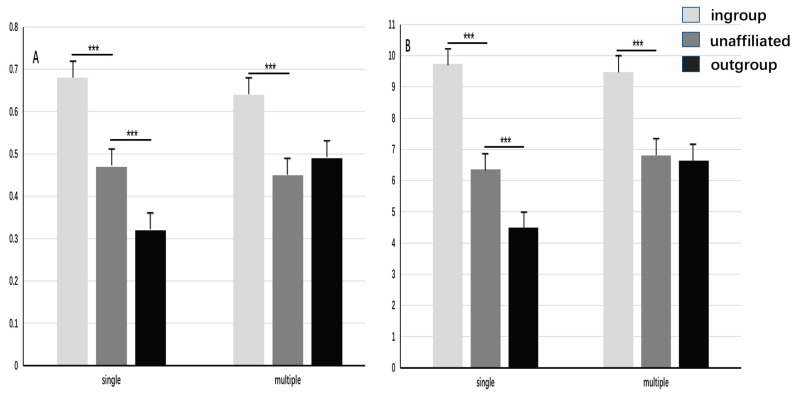
Impact of social identity complexity on intergroup bias in third-party fairness compensation. Note. (**A**) = compensation frequency. (**B**) = compensation amount. *** *p* < 0.001. The error bars are standard errors.

We used a repeated measure ANOVA of 3 (recipient group identity: ingroup, outgroup, unaffiliated; within) × 2 (social identity complexity: single, multiple; between) to compare the compensation frequency. Compensation frequency was calculated by dividing compensation times by 12 [28,29] (see Table 2). The results showed that the main effect of recipient group identity was significant: *F* (2,83) = 102.90, *p* < 0.001, η^2^p = 0.56; the compensation frequency for the ingroup recipient was significantly higher than that for the unaffiliated recipient (*p* < 0.001); and the latter was significantly higher than that for the outgroup recipient (*p* < 0.001). The main effect of social identity complexity was not significant: *F* (1,83) = 1.61, *p* = 0.208. The interaction effect was significant: *F* (2,83) = 9.61, *p* < 0.001, η^2^p = 0.18. The simple effect test showed that with a single identity, the compensation frequency for the ingroup recipient was significantly higher than that for the unaffiliated recipient and outgroup recipient (*p*_s_ < 0.001), and the compensation frequency for the unaffiliated recipient was significantly higher than that for the outgroup recipient (*p* < 0.001). With multiple identities, the compensation frequency for the ingroup recipient remained significantly higher than that for the unaffiliated and outgroup dictators (*p*_s_ < 0.001); however, the latter difference between the two was no longer significant (*p* = 0.39; see Figure 3).

We used the same method of repeated measures ANOVA to compare the compensation amount, which was calculated by dividing the number of tokens used to compensate by 12 [28,29] (see Table 2). The results showed that the main effect of recipient group identity was significant: *F* (2,83) = 81.07, *p* < 0.001, η^2^p = 0.49; the average compensation amount for the ingroup recipient was significantly higher than that for the unaffiliated recipient; and the latter was significantly higher than for the outgroup recipient (*p*_s_ < 0.001). The main effect of social identity complexity was not significant: *F* (1,83) = 2.85, *p* = 0.095. The interaction effect was significant: *F* (2,83) = 7.06, *p* < 0.001, η^2^p = 0.08. The simple effect test showed that with a single identity, the average compensation amount for the ingroup recipient was significantly higher than that for the unaffiliated and outgroup recipients (*p*_s_ < 0.001), and the average compensation amount for the unaffiliated recipient was significantly higher than that for the outgroup recipient (*p* < 0.001); however, the latter difference between the two was not significant (*p* = 0.97, see Figure 3).

The results of Experiment Two revealed that the compensation to the ingroup recipient was more than that to the unaffiliated recipient, and the compensation to the unaffiliated was more than that to the outgroup recipient. The former was a manifestation of ingroup love, and the latter was a manifestation of outgroup hate. Moreover, the results demonstrated that multiple social identities enhanced compensation for the outgroup recipient but not for the ingroup recipient. This result indicated that multiple identities could reduce outgroup hate but not ingroup love. However, with multiple identities, the compensation to ingroup members remained higher than that to outgroup members. This finding verified the conclusions of Experiment One that the intergroup bias could be reduced by multiple identities by reducing outgroup hate rather than ingroup love; however, it could not be eliminated completely.

## 4. General Discussion

Intergroup bias includes two components: ingroup love and outgroup hate. Although intergroup bias in third-party fairness maintenance has been widely discussed, few studies focused on ways to eliminate the bias. This study included ingroup and outgroup members and unaffiliated participants to separate the two components. We manipulated the social identity complexity of the two sides of the unfair event to affect intergroup bias. The results demonstrated that multiple identities reduced intergroup bias in third-party fairness maintenance by reducing outgroup hate rather than ingroup love.

### 4.1. Impact of Social Identity Complexity on Intergroup Bias

Intergroup bias widely exists in human social interaction behavior. Previous studies have shown that social identity complexity alleviates intergroup bias [25,26,30,31]. This study extended this effect to third-party fairness maintenance. Brewer notes that observing the origin of intergroup boundaries from the perspective of evolution sheds light on why social identity complexity affects intergroup attitudes [13]. Brewer argued that in the long process of evolution, humans gradually formed a cluster adaptation mechanism that makes people interdependent. However, not all people are worth relying on. Individuals choose people similar to themselves to form alliances. Those who do not conform to ingroup characteristics are regarded as outgroup members, and intergroup boundaries are created. However, the group environments in which people live are constantly changing, and intergroup boundaries are also changing. Therefore, Brewer proposed the optimal distinctiveness model of social identity, which considers that the intergroup boundary results from the interaction of people’s assimilation and dissimilation needs [32]. When people feel isolated and separated from others, their need for assimilation is stimulated, and they become more inclusive. When people feel that they are being submerged in the group, their alienation needs are stimulated, and they become more exclusive. Thus, group boundaries result from the balance between the two forces of individual inclusion and exclusion. When the original state of equilibrium is broken, the intergroup boundary changes.

The reason social identity complexity modifies third-party intergroup attitudes is that it can affect the equilibrium between inclusion and exclusion. Affected by multiple identities, the third- party’s attention to the group identities of the game players is weakened, and its attention turns to the multiple identities of the players. Paying attention to the multiple identities of others may cause identity overlap; outgroup members may belong with ingroup members because of a certain identity, which blurs intergroup boundaries. This blurring may have four specific effects [33]: (1) the assimilation process to the internal group and alienation process to the external group offset each other, decreasing the intergroup differences and destroying the cognitive basis of intergroup bias; (2) the significance of intergroup comparison for individual self-evaluation is reduced, and the motivation basis of intergroup deviation is destroyed; (3) the importance of any single identity to meet the individual’s group belonging needs and self-definition needs is reduced, which destroys the motivation basis of intergroup bias; and (4) if others belong to an ingroup in one identity but belong to an outgroup in another identity, the individual’s cognitive balance is destroyed. To re-establish the balance, individuals shorten the distance to outgroup members and reduce intergroup bias. Under the function of these four mechanisms, the foundation of outgroup hate is destroyed, which reduces the intergroup bias of third-party fairness maintenance [34].

Although multiple identities can reduce intergroup bias, the bias cannot be eliminated, as it comprises ingroup love and outgroup hate. Multiple identities can reduce outgroup hate but do not affect ingroup love. Therefore, intergroup bias cannot be eliminated entirely. Despite this effect, multiple identities are meaningful in promoting intergroup relations, as multiple identities can make people treat outgroup and unaffiliated members equally. This treatment reduces the exclusion and hostility to external groups, thus reducing intergroup conflicts.

### 4.2. Optimization of Third-Party Fairness Maintenance

Third-party fairness maintenance can become an important fairness maintenance mechanism because it offers objective neutrality; however, intergroup deviation damages this objective neutrality. Therefore, controlling intergroup bias is necessary to optimize third-party fairness maintenance. To achieve this goal, many methods, such as avoidance and blind evaluation systems, have been proposed in social governance. Although these methods can reduce intergroup bias in third-party fairness maintenance to a certain extent, some limitations to this process remain. These methods are not based on psychological means but have certain administrative attributes, which restrict people’s freedom of action and increase the cost of social governance. Moreover, these methods eliminate intergroup bias by shielding group relations and identity information of the parties concerned; however, in real life, identity information and group relations formed by identity information are ubiquitous, and only some can be shielded. In addition, intergroup bias has a certain secrecy; that is, its implicit form exists in people’s psychological activities, which cannot be shielded.

Social identity complexity can overcome the above limitations to some extent. Social identity complexity affects intergroup bias by presenting information regarding multiple identities. It uses identity information rather than shielding identity information; therefore, it is more widely applicable than administrative means and affects people’s implicit psychological activities. In addition, social identity complexity is a method and strategy based on psychology, which has low dependence on administrative power and does not restrict people’s freedom of choice. The increased governance costs are also limited, which makes the strategy of using social identity complexity more advantageous [35]. It can provide effective reference for optimizing the third-party fairness maintenance mechanism.

### 4.3. Relationship between Ingroup Love and Outgroup Hate

Many studies have regarded ingroup love and outgroup hate as the same thing, believing they are different sides of the coin [8,11,12]. However, this study found that multiple identities can affect outgroup hate but not ingroup love, which indicates that the two components are not completely consistent. This finding provides a new horizon and a relatively optimistic expectation to observe intergroup fairness; that is, the construction of ingroups is not necessarily related to the outgroup damage.

As early as 1954, Allport noted that an individual’s attachment to the internal group does not equate with hostility and attack on an external group [20]. The field research conducted by Brewer and Campbell in East Africa confirms this viewpoint [21]. The study of the intergroup prisoner game showed that whether the two components could be separated in research methods directly affects observation of the relationship between them [16,17,18,19]. The difference of research methods reflects a different understanding of intergroup competition. If the intergroup competition is understood as the competition of different groups for the same goal, the two components are not separated in research methods, and it is inevitable to observe that the two components as accompanied by each other. In contrast, if the intergroup competition is understood as different groups pursuing their own goals, the two components are separated in research methods, and it is possible to observe that the two components are not necessarily accompanied.

The phenomenon that social identity complexity only affects outgroup hate but not ingroup love may also be related to cultural factors. Collectivist culture focuses on the loyalty of individuals to internal groups, which enhances ingroup love [36]. The Oriental culture has a nature of collectivism. This study was conducted in China, which has an oriental culture, may enhance ingroup love and will not be weakened by multiple identities.

## 5. Conclusions

Social identity complexity of the two sides of unfair events reduced intergroup bias in third-party fairness maintenance by reducing outgroup hate rather than ingroup love. Specifically, multiple identities reduced third-party punishment for outgroup violators and improved third-party compensation for outgroup victims; however, multiple identities did not affect the punishment for ingroup violators and compensation for outgroup victims. This indicated that ingroup love and outgroup hate in third-party fairness maintenance were two independent mental mechanisms. These findings provide useful insights into understanding intergroup bias and promoting intergroup fairness and social harmony.

## Figures and Tables

**Table 1:** Punishment frequency and punishment amount *(M ± SD)*.

Social Identity Complexity	Dictator’s Group Identity	Punishment Frequency	Punishment Amount
single	ingroup	0.28 ± 0.18	3.85 ± 2.62
	unaffiliated	0.64 ± 0.18	8.18 ± 2.78
	outgroup	0.76 ± 0.14	10.64 ± 2.17
multiple	ingroup	0.30 ± 0.19	4.00 ± 2.36
	unaffiliated	0.67 ± 0.18	8.43 ± 2.33
	outgroup	0.67 ± 0.20	8.56 ± 2.33

**Table 2:** Compensation frequency and compensation amount *(M ± SD)*.

Social Identity Complexity	Recipient’s Group Identity	Compensation Frequency	Compensation Amount
single	ingroup	0.68 ± 0.17	9.73 ± 2.92
	unaffiliated	0.47 ± 0.16	6.36 ± 2.34
	outgroup	0.32 ± 0.17	4.49 ± 2.44
multiple	ingroup	0.64 ± 0.17	9.48 ± 2.46
	unaffiliated	0.45 ± 0.19	6.81 ± 3.33
	outgroup	0.49 ± 0.18	6.64 ± 2.97

## Data Availability

The data could be downloaded from https://www.scidb.cn/anonymous/QVJWSlJu (accessed on 25 February 2022).
